# Experimental Validation of Deflections of Temporary Excavation Support Plates with the Use of 3D Modelling

**DOI:** 10.3390/ma15144856

**Published:** 2022-07-12

**Authors:** Kopras Marek, Buczkowski Wiesław, Szymczak-Graczyk Anna, Walczak Zbigniew, Gogolik Sławomir

**Affiliations:** 1 Research and Development Department, Kopras Sp. z o. o., Szklarnia 7, 64-510 Wronki, Poland; marek@kopras.pl; 2 Department of Construction and Geoengineering, Faculty of Environmental and Mechanical Engineering, Poznan University of Life Sciences, Piątkowska 94 E, 60-649 Poznań, Poland; wabucz@o2.pl (B.W.); zbigniew.walczak@up.poznan.pl (W.Z.); slawomir.gogolik@up.poznan.pl (G.S.)

**Keywords:** 3D scanning, excavation, excavation support, support plate, deflection arrow, finite difference method

## Abstract

Almost every project is accompanied by earthworks, very often involving various types of excavation, and the work of people in the excavations. One of the most important tasks in earthworks is to ensure that the walls of the excavation are protected against sliding and that people working in and around the excavation are safe. Very often, in addition to criteria relating to safety and stability of the excavation, economic considerations are also an important criterion. This issue arises as early as the design stage and is related to the choice of construction and materials of which the shoring is to be made in such a way as to be able to withstand the pressure of the soil, ground loads resulting from stored excavated material and the operation of working machinery. Ongoing monitoring of the excavations and their reinforcement is also very important. The paper describes the unique results of experimental field tests, the purpose of which was to analyse the values of deflections of steel support plates of temporary excavation carried out on the object in 1:1 scale. The course of the experiment is presented for excavation support plates with a total depth of 6 m. Direct tests of the deflection arrow were carried out using two techniques, traditionally with a patch, and with laser scanning. Field tests were carried out for the designed situation without backfill load as well as for backfill load of 3.84, 15.36, 26.88 and 38.4 kN·m^−2^, respectively, for two measurement stages. Stage-I of the study consisted in collecting the results for soil in intact condition, whereas Stage-II collected results for loosened soil. The research experiment was supported by numerical calculations performed using the finite difference method in variational approach. The measured maximum deflections ranged from 14.40 to 16 mm, and the calculated values were 14.95 and 14.99 mm. The comparison of calculation results and tests proved to be very consistent. The analysis of the values of deflections showed that backfill load does not have a significant effect on the deflection of the lower plate, but it does affect the deflection of the first plate up to a depth of 1.2 m. Based on the obtained results, it is recommended to assume the limit (maximum) deflection arrow for support plates of temporary excavations at least as *w_gr_* = *L*/130, where *L* is the span of the plate. The calculation of deflection values was based on deflection values obtained experimentally and numerically for two steel variants: S235JR and S355JR. The *w_gr_* indicator of the maximum deflection arrow proposed by the authors is not stipulated by the industry standards, but it can be very helpful for the designing of excavation reinforcement.

## 1. Introduction

Sustainable growth of societies and continuous expansion of technical infrastructure result in the emergence of new network, communication, industrial and developmental investments. Systematic increase in the value of land means that more and more structures and installations are located underground. Continuous, conscious, sustainable development is associated with the need to build various underground networks, both technological and the ones that result from the environment requirements related to the protection of soil, water and air against pollution. The construction of water and sewage networks involves performing earthworks and laying pipelines in various types of excavations. In engineering practice, there are several ways (methods) to protect excavation walls against sliding. These include tight walls made of steel sheet piles (Larssen profiles), Berlin walls, cavity walls (concrete) formed in the soil, palisades made of piles (i.e., CFA or micro-piles), column walls made by jet injection as well as reusable system supports [1,2,3]. In practice, medium-deep and deep rope excavations are not performed without supports, with slopes having inclination corresponding to the angle of internal friction of the soil, as this would require a large strip of land and significant earthworks. Securing excavation walls by making tight walls with the use of various types of steel sheet piles or making Berlin type walls is associated with mechanical drive piling (often impact) of sheet piles or steel profiles (I-sections) [4,5,6]. The resultant noise and vibrations can be burdensome for residents and pose a threat to the durability of buildings (occurrence of scratches, cracks, subsidence). Therefore, in inhabited areas, works that require a pile driver are impermissible. However, if this is the case, only engines that drive support elements statically into the soil should be used. The above-mentioned methods of securing excavation walls are not suitable for supporting long line excavations. Moreover, cavity walls (concrete) formed in the soil are not suitable for securing walls of temporary line excavations. To protect and secure excavation walls, reusable system supports are used, for instance, those produced by KOPRAS, the Polish company. The components of these supports are poles, struts and plates of a steel or aluminum structure placed between poles, which must safely withstand and transmit the soil pressure acting on them. In general, plates are the most loaded elements of supports—their bearing capacity is determined by the structure, span and depth of excavations and the value of soil pressure. Plates must be constructed in such a way that they can transfer the soil pressure, and sometimes also the backfill load resulting from stored excavated material and operation of working machines (excavators, means of transport, cranes, etc.). The value of soil pressure depends on the condition and type of soil, the depth of excavations, the displacement of excavation supports, the backfill load, as well as the groundwater table. Properly designed support plates must ensure the safety of people’s work in any soil and water conditions in connection with the permissible excavation depth for a given type of plate, and they also affect the economic side of investments (production costs, transport, type of excavators used in drive piling due to the weight of plates). In order to be able to properly design excavation support plates, it is necessary to know the effective soil load, its distribution and the amount of pressure, and also the static diagram corresponding to the actual work of the structure should be adopted in calculations [7,8]. Earth pressure is related to the form and size of the displacement of the retaining structures in relation to the soil. The problem of earth pressure has been a frequent subject of scientific studies. Most often they take the form of numerical solutions [9] or model tests [10,11].

The numerical analysis of plates can be successfully performed using the finite difference method in variational approach. The literature on the subject contains numerous fundamental works on the finite difference method [12,13,14,15,16,17,18,19,20,21,22,23,24,25] and publications in which this method was applied and positively verified for calculating plates [26,27,28], tanks [29,30,31,32], shell structures and others [33,34]. Experimental verification of performed numerical calculations plays a key role in contemporary design. Implementation of a research model in 1:1 scale is a unique and undoubtedly the best way to verify numerical calculations, and non-invasive methods of measuring deflections and displacements of structural components are excellent measuring tools.

Validation of the obtained deflection results based on the finite difference method was carried out using a concrete tank model with an innovative measurement tool: a coordinate measuring arm with a touch probe [29]. Validation of calculations with the help of the coordinate measurement arm provided an opportunity to confirm their correctness. The coherence achieved is evidence in favour of the appropriateness of the applied calculation methods, i.e., the finite element method, which provided a basis for calculations using Autodesk Robot Structural Analysis Professional, as well as the finite difference method in terms of energy used to make traditional calculations [29]. Terrestrial Laser Scanning (TLS) [35] is a non-invasive, non-contact technique that enables fast and, what is very important, precise acquisition of data on the geometry of measured objects in the form of coordinates of points x, y, z. 3D laser scanning is currently used in many practical engineering applications, including inventory [36,37,38,39,40,41,42,43], inspection [44,45], verification of test results [29] and maintenance of buildings [46], particularly historical buildings (constituting the cultural heritage). Inventory can be carried out faster and more accurately than when using traditional solutions. Thanks to the use of TLS techniques, it is possible to obtain more complete data on the geometry of measured objects. It can be assumed that the data are continuous, not point-based—as it is in the case of classical solutions. The accuracy of models created with the use of TLS may vary from a few millimetres [41,47,48,49] to even decimetres [50]. The accuracy of measurements depends on several factors, including the distance and angle between the scanner and objects being scanned as well as the type of scanned surface, particularly in the case of highly reflective, mirror surfaces [51,52,53]. The advantages of TLS also include the possibility of obtaining full, three-dimensional information on objects in the form of measurement points, which are their geometric representation, being in fact a dense quasi-continuous cloud of points. The information obtained in this way, the cloud of points, can then be processed, generating i.e., orthophotos, CAD or BIM models, mesh models, models for visualisation, virtual walks, etc. In addition to numerous advantages of TLS, there are also several factors that can make it significantly difficult or even impossible to obtain high-quality data by means of laser scanning. Key issues to mention here are, among others, situations in which some parts of the scanned elements may be invisible, obscured from laser scanning. Moreover, glass elements (windows, mirrors), as well as wet or damp surfaces or surfaces with water on the surface, can cause disturbances. One of the major problems at the stage of further data processing may be the fact that an enormous data set is created, which will require large hardware and computing power for further computing (high requirements for hardware of computer systems).

Problems related to obscuring the visibility of scanned items can be solved via applying techniques combining laser measurements with manual measurements [54], as well as photogrammetry [55,56,57,58,59,60]. Fawzy [57] indicates that the combination of TLS techniques and short range photogrammetry increases the accuracy of models. Based on the measurements of 20 points, 10 lines and 6 control angles, Fawzy indicates that the maximum improvement in the quality of the model was 80.1, 66.4, and 84.2% for points, lines and angles, respectively [57].

TLS is also widely used for renovation and revitalization of historic buildings for which it is planned to build a BIM model or Heritage or Historic Building Information Modelling (HBIM). As a result, it is possible to build a 3D model to recreate the plans of historic buildings for which they did not exist, use it during planning of modernisation, revitalisation, but also operation or even visualisation of the object [56,61,62,63,64,65,66]. The use of TLS can also be helpful in assessing the technical condition of facilities [67]. This can significantly facilitate damage assessment and help in proposing an appropriate renovation system [45]. The accuracy of point clouds is sufficient to perform surface regularity checks.

The purpose of the work was to experimentally verify the values of deflections occurring in support plates subjected to soil pressure and backfill load in real conditions reflecting the work of the structure. The results of the measurements obtained based on the natural-scale experiment were compared with the values calculated with the finite difference method. The paper presents the test results of actual deformation of open excavation support plates depending on the acting backfill load. Since neither in the literature nor in the standards is there a deflection limit value for such structures as temporary excavation support plates, the paper includes the recommendation for the deflection arrow value, which can be used to calculate the maximum deflection in this type of construction facilities.

The conducted tests, measurements and calculations can be used to verify existing solutions and improve the structure of excavation support plates in order to ensure greater safety of manufactured and used products, as well as to reduce their cost.

## 2. Materials and Methods

### 2.1. Description of Research Location and Objectives

**Figure 1:**
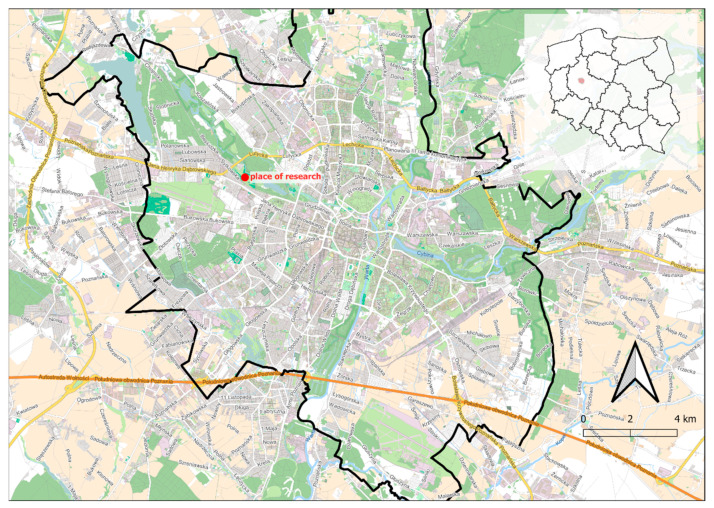
Location of the test stand (Poland, Greater Poland Voivodeship, the city of Poznań).

**Figure 2:**
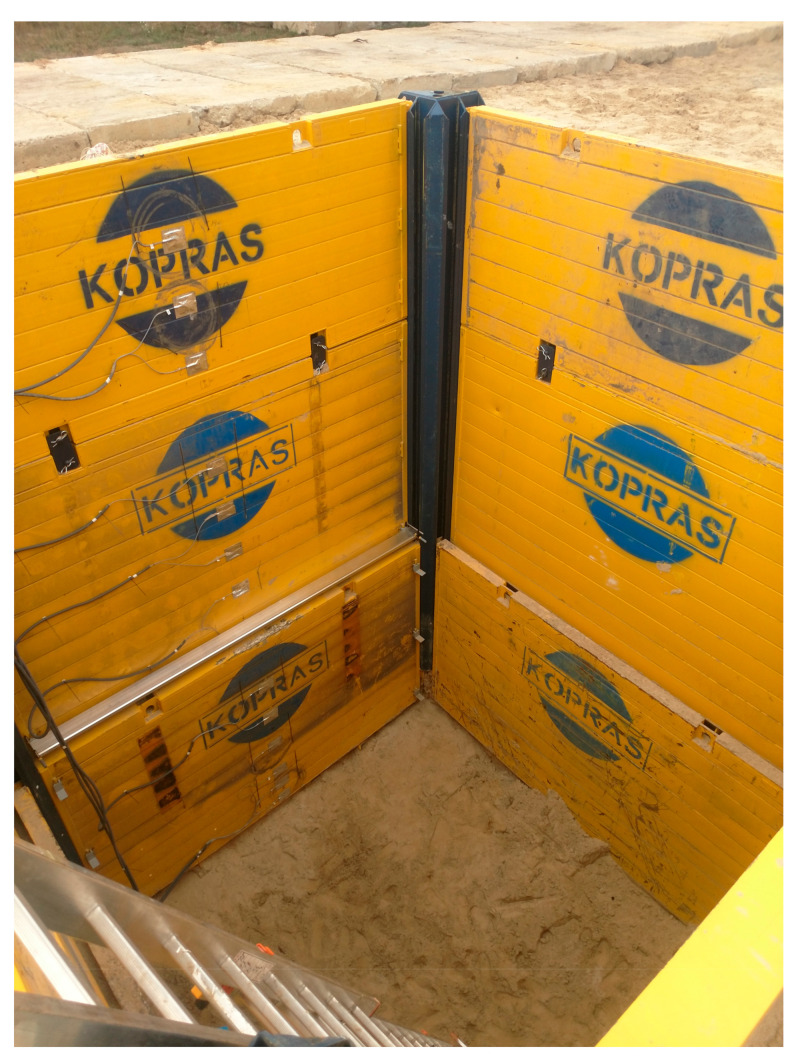
Test stand—view of the excavation and the excavation support.

The field tests were conducted from 4 September to 27 November 2019. The values of actual deformations of open excavation support plates were analysed depending on acting backfill load. The test stand was in a place with non-cohesive soils of a possibly homogeneous profile, with the groundwater table below the bottom of planned excavations. The area near Lake Rusałka was selected for the location of the test stand (Figure 1). The terrain where the test stand was installed was situated several meters above the water level. Before the final selection of its location, six boreholes were made in the area in question, from which soil samples were collected (with natural graining NU and natural humidity NW) in order to conduct laboratory tests and sounding with a static CPTU probe. These activities were aimed at checking the soil profile and groundwater level. Based on the measurements, the final location of the test stand was selected. It was a place where the groundwater table was not drilled, with convenient access and sufficient space for all operations related to excavation works and loading the backfill. In the selected place, an open excavation was made with dimensions 4 × 4 m, 6 m deep, reinforced with the system of steel plates (Figure 2).

**Figure 3:**
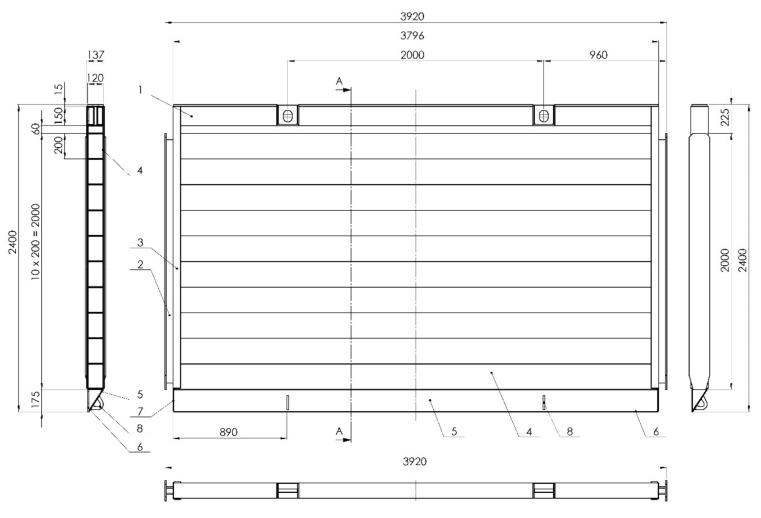
View and cross-sections of the support plate. Denominations: 1—plate head, 2—plate fin, 3—closing C-profile, 4—rectangular profile 200 × 10 × 4, 3760 mm long, 5—steel cutting edge g = 6 mm, 6, 7—closing sheet, 8—transport eye. Units [mm].

The excavation was secured as a point box composed of steel corner posts and a system of fin plates. The fin plate with dimensions 3.92 × 2.40 × 0.12 m was a welded structure made of square and rectangular sections and flat bars. The top horizontal edge, i.e., plate head, was a structure consisting of rectangular and square profiles, additionally reinforced with an overlay made of a 15 mm thick flat bar. The flat bar stiffened the top edge of the plate, preventing it from being dented by an excavator bucket during driving the plate. The bottom horizontal edge was a cutting section, which penetrated the excavation soil under the self-weight of the plate. All profiles on the left and right side of the plate were closed with vertical edges. A special C-profile, pulled on the fronts of horizontal profiles, had welded fins guiding the plate in the guides of the corner posts. Fins transmitted the response of a freely supported beam (which a fin plate is by definition) onto the supports. Each plate was strengthened by corner posts with sliding guides (Figure 3).

### 2.2. Geotechnical Measurements

During preliminary geotechnical tests, six test boreholes were made to verify the soil profile and groundwater level, which were the basis for selecting the optimal location of the test stand. Detailed geotechnical tests were carried out in the selected location, including drilling to a depth of 6 m, during which samples of soil with natural graining (NU) and natural moisture (NW) were taken for laboratory tests to determine their granulometric composition and natural moisture content. Soundings with a static CPTU probe were also performed. Static sounding tests were performed with a CPTU piezocone in accordance with the TC-16 procedure recommended by the International Society for Soil Mechanics and Geotechnical Engineering [68]. The tests used a standard measuring tip with a diameter of 36 mm, a base area 10 cm^2^, an apex angle 60° and a friction sleeve area 150 cm^2^. The main test consisted in pressing the measuring tip into the soil with a constant value equal to 2 cm·s^−1^. During penetration, three test parameters were recorded every two centimetres of depth increment: resistance of the cone—ci, friction on the friction sleeve area—fs pressure of excess water in pores—$u_{2}$. It allowed for determining soil characteristics in the following respects: separation of geotechnical layers with the determination of soil types; identification of the stress level in the soil; determination of the compaction degree (ID); determination of the shear strength parameter—the effective angle of internal friction—(*ϕ*′); determination of the soil deformability parameter—edometric compressibility modulus (*M_o_*).

### 2.3. Tests of Stresses in the Soil Using a Hydraulic Probe

Several days before the excavation was made and the support was lowered, hydraulic sensors for measuring soil pressure were inserted into the soil. The probe was inserted into the soil using the same technology as when performing CPTU probing and with the same device. The lowest sensor was at 6.0 m below ground level, and the remaining sensors at 4.5, 3.5, 2.5, 1.5, and 0.5 m below ground level. The measurements of soil pressure taken on 4 and 16 September 2019 corresponded to the resting pressure, as assembly of the support and the excavation had not started yet.

### 2.4. Soil Stress Tests Using a Hydraulic Probe

**Figure 4:**
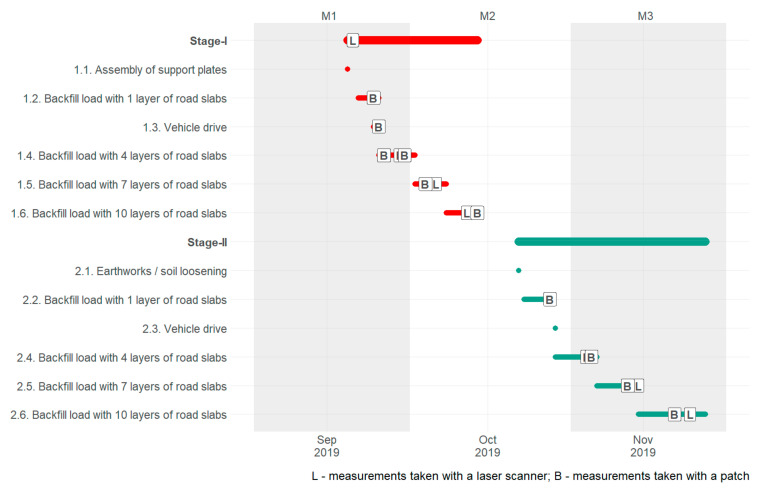
Scheme of tests (L—measurements taken with a laser scanner, B—measurements taken with a patch).

**Figure 5:**
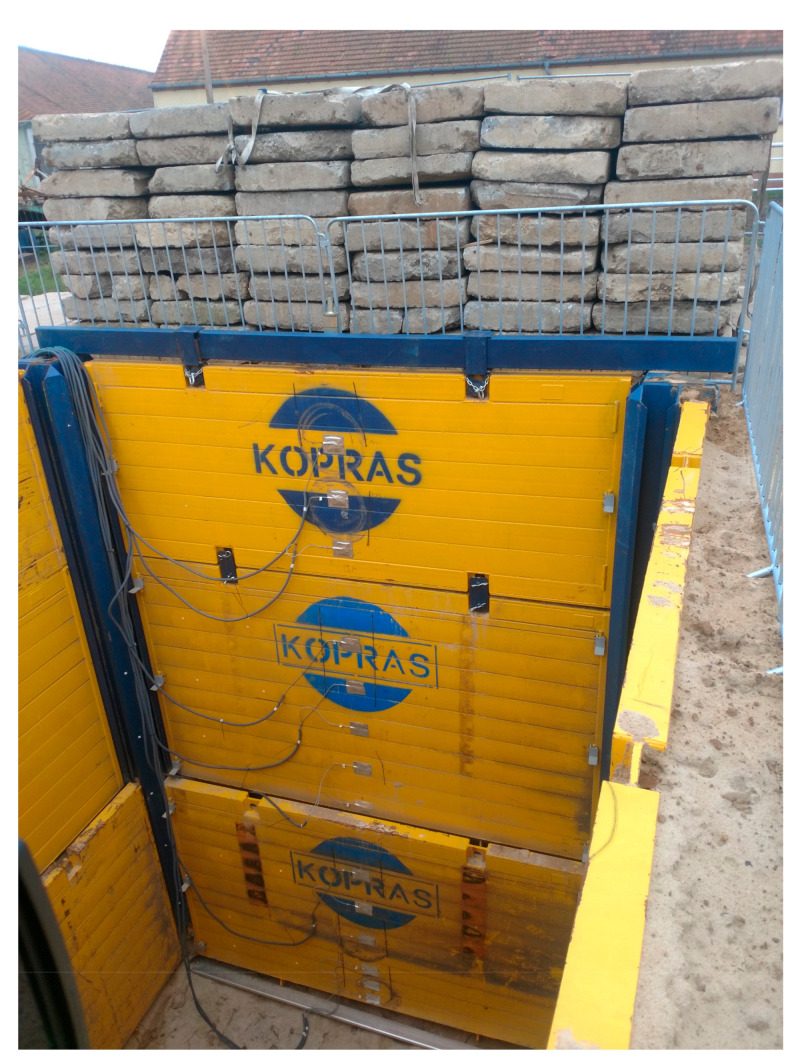
Loading of the backfill with 10 layers of road slabs (38.4 kN·m^−2^).

Tests were divided into two stages; the work scheme is presented in Figure 4. In the first stage (Stage-I), after the excavation was made and the walls were secured with plates, the backfill was loaded by adding one layer of 2.95 × 0.8 × 0.16 m road slabs, placed near the excavation. The soil around the excavation remained intact. Then, the backfill was additionally loaded using a 40 t vehicle, simulating the volume of traffic that usually takes place on construction sites. Next, the backfill was weighed down with slabs, placed successively in 4, 7 and 10 layers (Figure 5), resulting in obtaining the values of backfill load of 3.84, 15.36, 26.88 and 38.4 kN m^−2^, respectively. After each successive loading, the deflection arrow was measured with a patch and with the use of laser scanning.

The next stage (Stage-II) involved analysing the loosened soil. To change the condition of soil and its loosening, after removing the backfill material, the plates on the side of the tested wall were excavated within the fragmentation wedge, changing the parameters that affected soil pressure. Then, the excavation was backfilled, and the soil was compacted with layers of max. 50 cm. using a plate vibrator. Sounding with a CPTU static probe was carried out to determine the parameters of the backfill material.

### 2.5. Measurement of the Deflection Arrow Taken with a Patch

**Figure 6:**
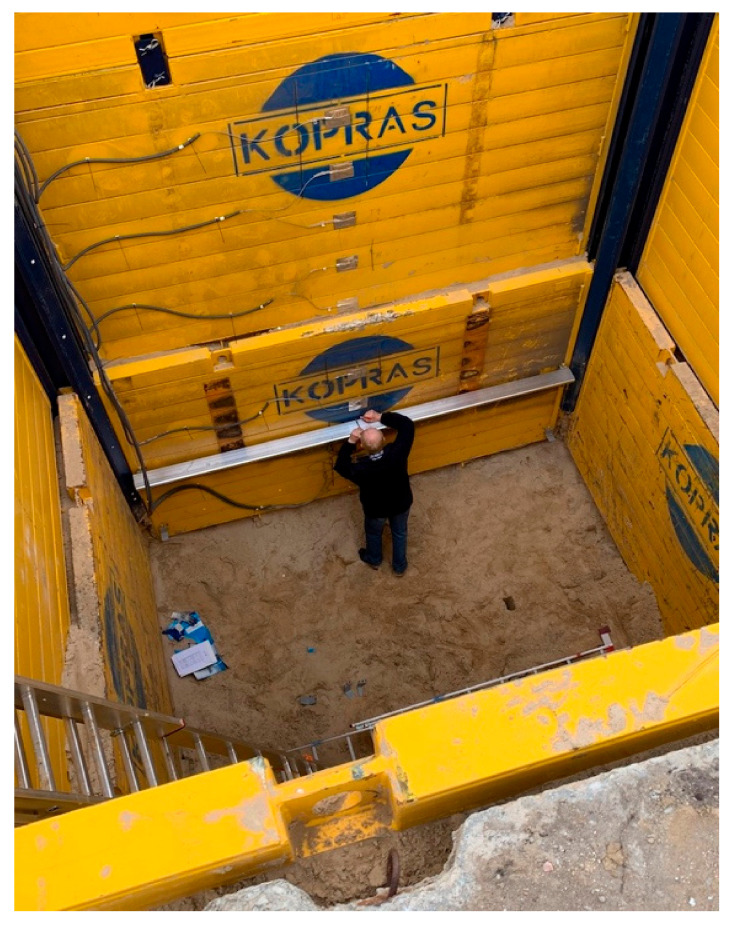
Direct measurement of the deflection arrow taken with a measurement patch.

The direct measurement of the deflection arrow was performed using a measurement patch (Figure 6). The patch had previously been calibrated for its straightness in the laboratory.

Measurements of the deflection arrow taken with a measurement patch were always made at the same, seven levels (Table 1), each time after changing the value of the backfill load by means of road slabs. They were then compared with the results obtained from laser scanning.

### 2.6. TLS Measurement

Terrestrial Laser Scanning is a technique that uses laser light for measurements. The measured object is illuminated by the scanner with a beam, which then, reflected from the object, returns to the scanner. The measurement of distance consists in measuring the change of the phase between the emitted and reflected beam (phase scanners) or in measuring the time it takes for the beam to travel back and forth (pulse scanners). Phase scanners, more commonly used nowadays, allow the registration of up to about 1,200,000 measurement points per second. By knowing the distance (*L*) from the scanner and the i-th measurement point, vertical angle (*β*) and horizontal angle (*α*) coordinates of a point in a 3D coordinate system in real time can be calculated using the following equation:

(1) $$\left\{\begin{array}{c} X_{i}=L\;\cos\beta \cos\alpha \\ Y_{i}=L\;\cos\beta \sin\alpha \\ Z_{i}=L\;\sin\beta \; \end{array}\right.$$

Eight scanning series were performed, four scans for each stage with the use of a 3D scanner Surphaser 100HSX from Surphaser, Redmond, WA, USA. The manufacturer states that the max. accuracy of the distance measurement is ±0.3 mm and the angular resolution is equal to 1 arcsec. The laser wavelength is 685 nm.

The first scan represents the deformation of the excavation support not subjected to an additional load, which is called the reference scan made to calibrate the device.

Table 2 summarises the dates of scanning measurements for Stages-I and Stage-II with the value of backfill load.

**Figure 7:**
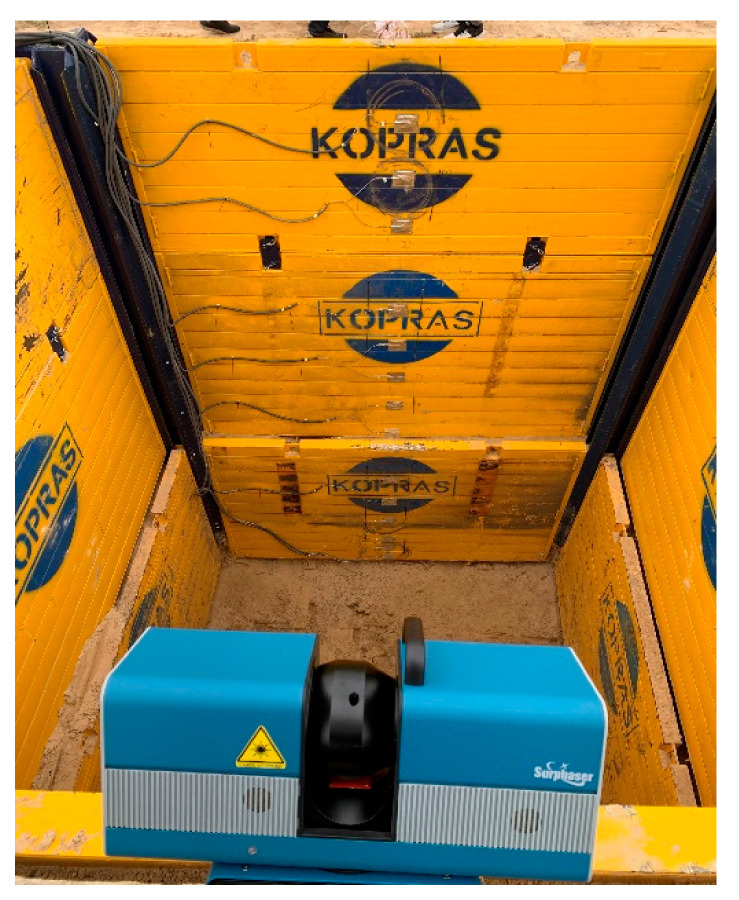
Location of the scanner.

The first scan took place on 20 September 2019, right after the excavation was made and strengthened with steel support plates. Scanning was performed in such a way that the laser beam reached the bottom of the excavation (Figure 7). The initial processing of point clouds related to the registration, filtration and removal of the unnecessary data was carried out in SurphExpress, the manufacturer’s software, and then data sets were analysed in the open-source CloudCompare environment in order to estimate deformations.

**Figure 8:**
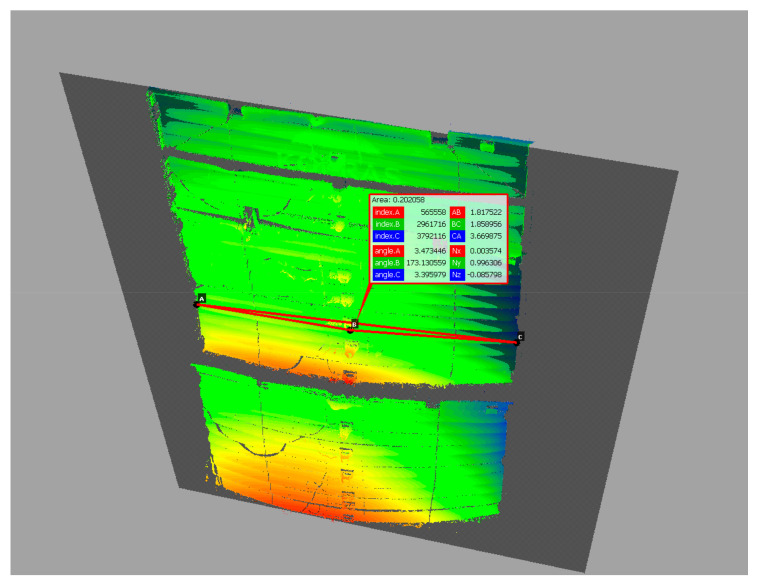
Measurement of the deflection arrow.

Subsequent scans were performed each time when the soil was loaded with concrete plates near the excavation (Figure 5). After each scan, a point cloud was created in SurphExpress, which was then exported to CloudCompare, where its further processing and analysis was carried out. Filtered point clouds, after uploading into the program, were aligned to the reference plane, obtaining the distribution of displacements. The deflection arrow value was determined by measuring the basic lengths according to the scheme in Figure 8. Length A-B (half plate width) and angle CAB were read. The value of the deflection arrow was determined from trigonometric relationships for the ordinates corresponding to manual measurements taken with a measurement patch.

Then, the distribution of the differences of the point clouds between the reference model and the analysed point cloud was estimated. The Distance Cloud2Cloud tool of CloudCompare was used. The distances between the two-point clouds were computed as the ‘nearest neighbour distance’: for each point of the compared cloud, CloudCompare searches the nearest point in the reference cloud and computes their (euclidean) distance. However, because the points in the two point clouds do not exactly correspond to each other, a better estimate of the distance from each point of the compared cloud to its nearest point in the reference cloud can be obtained by replacing the distance from the local mathematical model in the ‘nearest’ point and several of its surrounding neighbours represented as a local plane [69]. This is statistically more precise and less dependent on the cloud sampling. Quadric local model (Height function) was used. In fact, the corresponding model is a quadratic function (6 parameters: Z = a.X^2^ + b.X + c.XY + d.Y + e.Y^2^ + f).

### 2.7. Static and Strength Calculations of the Support Plate for the Plate System Calculated with the Finite Difference Method

Calculations were made using the finite difference method in the variational approach.

The linear algebraic equations, from which the desired deflections at the nodes of the subdivision grid will be determined, are obtained in the condition for the minimum of the functional:

(2) $$\frac{\partial V}{\partial w_{k}}=0$$

where for each *w_k_*, where *k* is the number of meshes of the subdivision mesh. The above condition (2) follows from the theorem that:

“If the system is in steady state equilibrium, its total energy reaches a minimum. Applying this statement to the analysis of plate bending, we must take into account that the total energy in such cases consists of two parts: the elastic energy of bending and the potential energy of the load, located on the plate” [16]. Thus, the problem of plate bending reduces in each particular case to finding such a function in (*x*, *y*) that would satisfy the given boundary conditions and give the smallest value of the integral described by the relation (3). The functional describing the total energy of elastic deformation for plate structures, also taking into account the ribs resting on the elastic foundation with potential temperature loads, taken from work [70], has the form:

(3) $$\begin{array}{cc} V=\;\frac{D}{2}\underset{A}{\iint }\{{\left(\frac{\partial^{2}w}{\partial x^{2}}\right)}^{2} & +2{\left(\frac{\partial^{2}w}{\partial x\partial y}\right)}^{2}+{\left(\frac{\partial^{2}w}{\partial y^{2}}\right)}^{2}+2v\left[\frac{\partial^{2}w}{\partial x^{2}}\frac{\partial^{2}w}{\partial y^{2}}-{\left(\frac{\partial^{2}w}{\partial x\partial y}\right)}^{2}\right]\\ & +2\left(1+v\right)\frac{\alpha_{t}\Delta T}{h}\left(\frac{\partial^{2}w}{\partial x^{2}}+\frac{\partial^{2}w}{\partial y^{2}}+\frac{\alpha_{t}\Delta T}{h}\right)\}dA++\frac{1}{2}\underset{A}{\iint }Kw^{2}dA-\underset{A}{\iint }qwdA\\ & +\frac{E_{z}J}{2}\overset{\;}{\underset{S}{\int }}{\left(\frac{\partial^{2}w}{\partial s^{2}}\right)}^{2}dS \end{array}$$

where:

*w*—plate deflection,
ν—Poisson’s ratio,
*D*—flexural rigidity ($D=\frac{Eh^{3}}{12\left(1-\nu^{2}\right)}$),
*h*—plate thickness,
*E*—elasticity modulus for the plate material,
*E_z_*—modulus of elasticity for the rib material,
*S*—rib area,
*s*—size of the mesh division used in calculation, *A*—plate area,
*q*—load perpendicular to the median plane of the plate,
*K*—subgrade reaction modulus,
*α_t_*—coefficient of linear thermal expansion of the plate material,
Δ*T* = *t_d_* − *t_g_*—temperature difference between the plate planes,
*J*—moment of inertia of the rib cross-section.

When starting the search for solutions for specific plate structures, the area *A* of the plate was divided by a discrete grid with meshes *s* × *s* into elementary sub-areas.

Further analysis was based on the adopted designations of the second derivative of the deflection function (4):

(4) $$w_{xx}^{2}={\left(\frac{\partial^{2}w}{\partial x^{2}}\right)}^{2}\;w_{xy}^{2}={\left(\frac{\partial^{2}w}{\partial x\partial y^{2}}\right)}^{2}w_{yy}^{2}={\left(\frac{\partial^{2}w}{\partial y^{2}}\right)}^{2}\;w_{ss}^{2}={\left(\frac{\partial^{2}w}{\partial s^{2}}\right)}^{2}\;w_{xx}=\frac{\partial^{2}w}{\partial x^{2}}\;\;\;w_{yy}=\frac{\partial^{2}w}{\partial y^{2\;}}\;$$

Expressing the partial derivatives of the deflection surface occurring in the energy functional through difference quotients, replacing the integration over the surface with summation over elementary sub-areas and assuming the Poisson’s ratio *ν* = 0 and not considering the temperature load, the functional describing the elastic deformation energy of the plate took the form:

(5) $$V=\frac{D}{2}\underset{A}{\iint }\left(w_{xx}^{2}+2w_{xy}^{2}+w_{yy}^{2}\right)+\frac{1}{2}\iint_{A}^{\;}Kw^{2}dA-\iint_{A}^{\;}qwdA+\frac{E_{z}J}{2}\int_{S}^{\;}w_{ss}^{2}dS\;\;$$

## 3. Results

### 3.1. Geotechnical Tests

The conducted geotechnical tests, both laboratory tests on NU and NW samples, and CPTU tests indicated that under the layer of soil, from about 0.2 m to about 1.8 m below ground level, there were mainly wet, medium-compacted medium sands (Medium Sand MSa), while below there were moist, compacted sands with admixtures of silty sand or dust fine sand (Fine Sand FSa). Table 3 shows the development of the geotechnical profile for Stage-I of the study.

**Figure 9:**
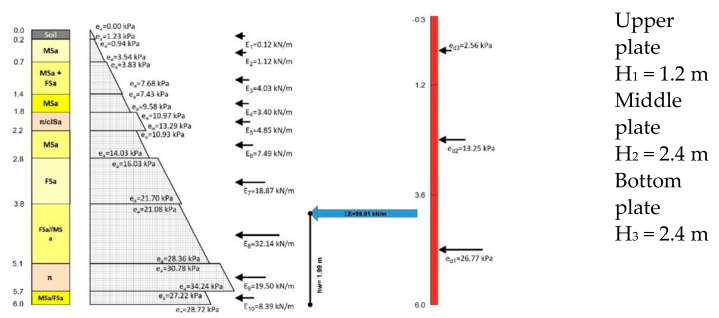
Diagram of computational soil pressure in the vicinity of the test stand for Stage-I.

Figure 9 shows the results of characteristic soil pressure in the vicinity of the test stand, calculated in accordance with recommendations provided in PN-81/B-03010 [72], assuming the geotechnical parameters provided in Table 3. Calculations were made without taking into account backfill load, but in compliance with recommendations of PN-83/B-03010 [72] for individual soil layers, the weight of the higher layers was treated as a substitute load of the backfill.

The assumed value of soil pressure corresponded to the total sum of pressures occurring in individual soil layers. For the unloaded backfill, the total value of soil pressure E_a_ was 99.91 kN m^−1^ (Figure 8), thus the resultant ordinate of soil pressure e_a_ located in the lower part of the plate was equal to 33.30 kN m^−2^

$$0.5\cdot e_{a}\cdot H=99.91\;\mathrm{kN}\cdot \to e_{a}=\frac{99.91}{0.5\cdot 6}=33.30\;\mathrm{kN}\cdot m^{-2\;}$$

### 3.2. Measurements of the Deflection Arrow Taken with a Patch

Manual measurement of the deflection arrow was performed each time in the morning hours before the work related to changing the load conditions started. The first measurement of the deflection arrow was taken on 24 September 2019. Table 4 summarizes the deflection arrow values taken with a measurement patch for Stage-I of the study, whereas Table 5 summarizes the deflection arrow values after changing the soil conditions (Stage-II).

The maximum value of the deflection arrow for Stage-I (15.5 mm) was read for the bottom plate of the excavation support. The measurement was taken at 5.72 m below ground level. After changing the soil conditions (Stage-II), as before, the maximum deflection arrow (11.0 mm) was recorded at 5.72 m below ground level.

### 3.3. Scanning

**Figure 10:**
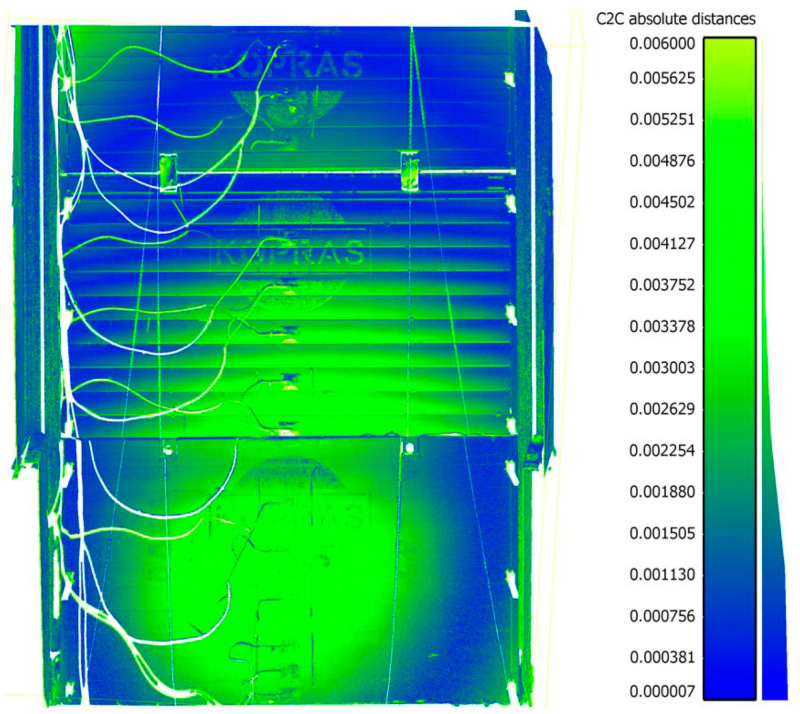
Deformation of excavation support plates on 14 November 2019, Stage-II, backfill load 26.88 kNm^−2^.

The first scans were carried out on 20 September 2019, right after the excavation was made and its walls were strengthened with support plates. The point cloud was assumed as referential, to which the value of the deformation of support plates was then estimated, by comparing the point clouds from the next scan performed after subsequent loading of the soil near the excavation area. Subsequent scans were performed in the same way as the first scan. Deformation distributions of excavation support plates were obtained by comparing two Cloud2Cloud point clouds in CloudCompare. Exemplary deformation distribution of excavation support plates as of 14 November 2019, for Stage-II and the value of the backfill load 26.88 kNm^−2^ is shown in Figure 10. Table 6 presents the measured values of the deflection arrow for Stage-I, whereas Table 7 includes the values for Stage-II. Measurements were taken at levels consistent with direct measurements using a measurement patch (Table 4 and Table 5).

### 3.4. Static and Strength Calculations for a Support Plate Freely Supported on Two Opposite Edges, Based on Tables

To verify the deflections obtained in situ, numerical calculations were performed using the finite difference method. First, verification calculations were made based on Design Tables [73], and second, a detailed numerical analysis was carried out, the results of which are provided in the next section of the paper.

**Figure 11:**
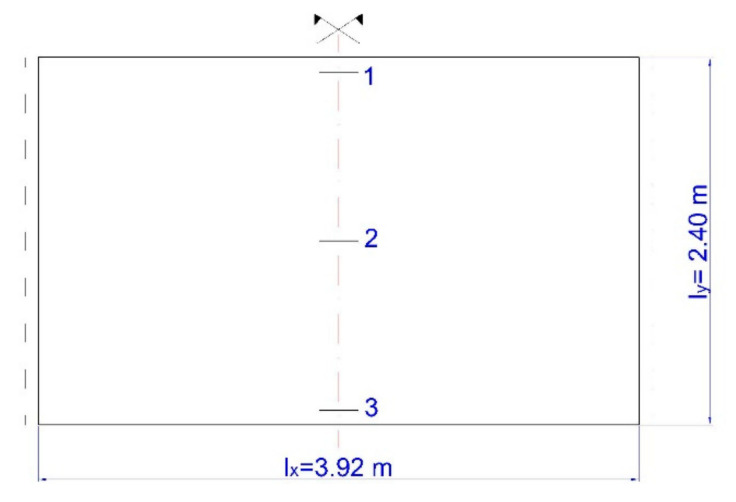
Diagram of the plate with dimensions 3920 × 2400 × 12 mm with marked points for which the deflections were calculated.

The structure of the plate consisted of closed rectangular steel profiles with dimensions 200 × 120 × 4 mm (Figure 3), the overall dimensions of the plate were 3920 × 2400 × 120 mm (Figure 11). The plate was subjected to a uniformly distributed load *q*_1_ and soil pressure *q*_2_.

By assuming the Young’s modulus of steel (also referred to as the modulus of elasticity for steel) as *E* = 210 GPa and the rigidity of the plate (ribs excluded) as $D=\frac{E\cdot \left(H^{3}-h^{3}\right)}{12}=\frac{210\cdot 10^{6}\cdot \left(0.12^{3}-0.112^{3}\right)}{12}≅5654\;\mathrm{kNm}$, based on Tables [73], the following value of hydrostatic thrust *q*_2_ was achieved:

$$\frac{l_{y}}{l_{x}}=\frac{2.40}{3.92}=0.61\;$$

$$w_{1}=0.00570\frac{q_{2}\cdot l_{x}^{4}}{D}=0.000238q_{2\;}$$

$$w_{2}=0.00652\frac{q_{2}\cdot l_{x}^{4}}{D}=0.000272q_{2\;}$$

$$w_{3}=0.00735\frac{q_{2}\cdot l_{x}^{4}}{D}=0.000307q_{2\;}$$

For an evenly distributed load (*q*_1_), the value of deflection *w*_1_ = *w*_2_ = *w*_3_ = *w*, was:

$$w=\frac{5}{384}\cdot \frac{q_{1}\cdot l_{x}^{4}}{D}=0.01302\cdot \frac{q_{1}\cdot l_{x}^{4}}{D}=0.000544q_{1}$$

**Figure 12:**
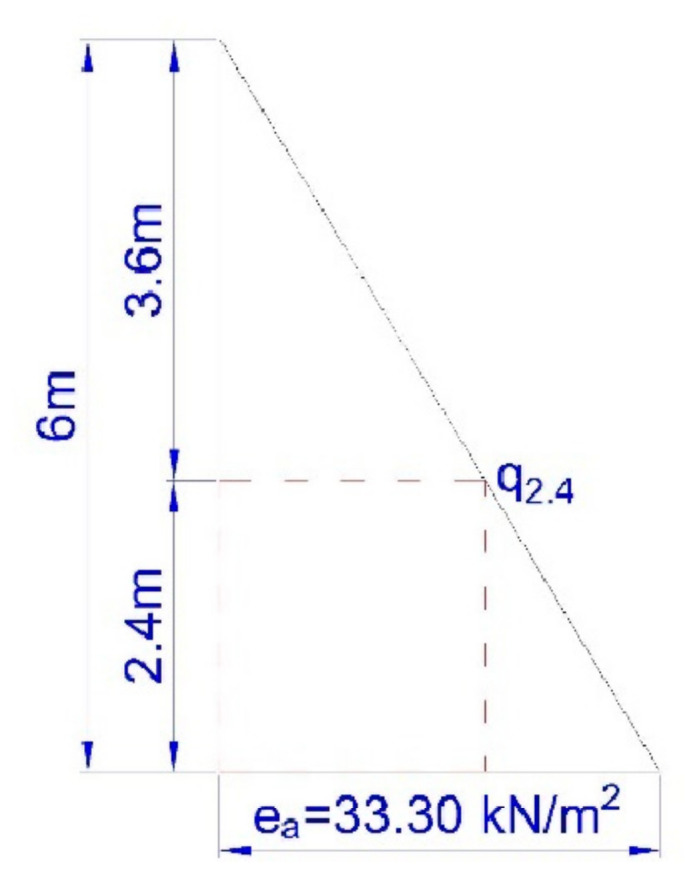
Diagram of the assumed value of soil pressure.

In order to estimate the values of deflections, Figure 12 shows the assumed value of soil pressure calculated for Stage-I (Figure 9).

$$\frac{33.30}{6}=\frac{q_{2.4}}{3.6}\;q_{2.4}=\frac{33.30\cdot 3.6}{6}=19.98\;\mathrm{kN}\cdot m^{-2\;}$$

Calculations considered the load acting on the bottom plate as $q_{1}=19.98\;\mathrm{kN}\cdot m^{-2}\;$(evenly distributed load) and $q_{2}=33.30-19.98=13.32\;\mathrm{kN}\cdot m^{-2}\;$(hydrostatic thrust).

For the above data, it was calculated:

$$w_{1}=0.000544\;\cdot q_{1}+0.000238\cdot q_{2}=14.03\;\mathrm{mm}\;\;w_{2}=0.000544\;\cdot q_{1}+0.000272\cdot q_{2}=14.49\;\mathrm{mm}\;\;w_{3}=0.000544\;\cdot q_{1}+0.0003070\cdot q_{2}=14.95\;\mathrm{mm}\;$$

### 3.5. Static and Strength Calculations of a Support Plate for the Plate System Using the Finite Difference Method

**Figure 13:**
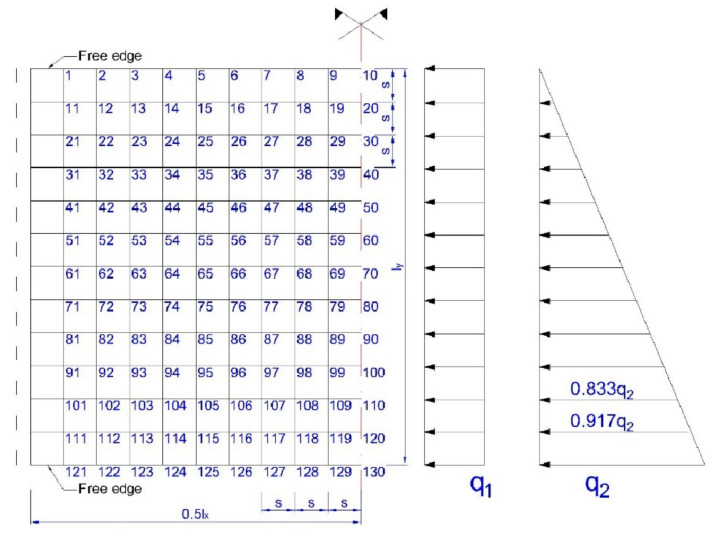
Adopted division mesh and load diagrams: evenly distributed load *q*_1_ and hydrostatic load *q*_2_.

For detailed calculations using the finite difference method, square meshes of the division grid (*s* = 20 cm) and a static plate system with two opposite free edges and two other freely supported edges were adopted. The adopted division mesh with the numbering of nodes is shown in Figure 13.

By solving the systems of equations with 130 unknowns for a uniformly distributed load and hydrostatic thrust, the coefficients proportional to deflections at individual nodes of the division mesh were obtained.

To obtain the values of deflection *w*(*q*_1_), the obtained coefficients should be multiplied by $\frac{q_{1}s^{4}}{D}$, and for the values of deflection *w*(*q*_2_) by $\frac{q_{2}s^{4}}{D}.$

To compare the values with the calculations given above, according to [73], the exact values of deflections at points 10, 70 and 130 were calculated (Figure 13) and they were:

for an evenly distributed load:

$$w_{10}=w_{70}=w_{130}=0.01305\frac{q_{1}\cdot l_{x}^{4}}{D}=0.000545q_{1\;}$$

and for hydrostatic load:

$$w_{10}=0.00570\frac{q_{2}\cdot l_{x}^{4}}{D}=0.000238q_{2\;}\;w_{70}=0.00653\frac{q_{2}\cdot l_{x}^{4}}{D}=0.000273q_{2\;}\;w_{130}=0.00737\frac{q_{2}\cdot l_{x}^{4}}{D}=0.000308q_{2\;}$$

By assuming $q_{1}=19.98\;\frac{\mathrm{kN}}{m^{2}}\;$(evenly distributed load) and $q_{2}=13.32\;\frac{\mathrm{kN}}{m^{2}}\;$(hydrostatic thrust), it was calculated:

$$w_{10}=0.000545\;\cdot \;q_{1}+0.000238\cdot q_{2}=14.06\;\mathrm{mm}\;\;w_{70}=0.000545\;\cdot \;q_{1}+0.000273\cdot q_{2}=14.52\;\mathrm{mm}\;\;w_{130}=0.000545\;\cdot \;q_{1}+0.000308\cdot q_{2}=14.99\;\mathrm{mm}\;$$

The comparison of results obtained with calculations based on Tables [73] and detailed calculation made with the finite difference method showed a very high consistence.

### 3.6. Calculations of the Deflection Arrow of the Plate

The serviceability limit state is the state beyond which a plate no longer meets its operational requirements. According to the guidelines of PN-EN 13331-1-1:2002 [74], the maximum deflection of plates and sliding guides should be certified with reference to the load equal to the immediate strength divided by partial factors of *γ_F_* = 1.5 (for soil action) and *γ_M_* = 1.1 (for strength).

The above recommendations correspond to the guidelines of PN-EN 1990:2004 [75], which for the serviceability limit states recommend taking partial factors with the value of *γ* = 1.

The literature on the subject and applicable standards do not specify the deflection limit for temporary excavation support plates. This is an inconvenience for site managers, as there is no value with which to compare the deflection of support plates, which can be easily measured with a patch at any construction site.

On the basis of the obtained deflection values for the lower edge of the plate for tabular [73] and detailed (FDM) solutions, an attempt was made to determine the permissible (limit) value of the deflection arrow, which was also compared with the values read from measurements taken with a patch and a laser scanner.

Deflection at Point 3 (Figure 11) was 14.95 mm, and at Point 130 (Figure 13) it was 14.99 mm. For the plate with the length *l_x_* = 3.92 m it took the value:

(6) $$\frac{3920}{14.95}=262\to \;w=\frac{l_{x}}{262}\;\mathrm{and}\;\frac{3920}{14.99}=261.5\to \;w=\frac{l_{x}}{261}.$$

Based on the actual measurements of deflections in situ (Table 4 and Table 6), the greatest value of the deflection arrow was 15.5 mm (patch) and 16 mm (scanner); for these values it was then designated:

$$\frac{3920}{15.5}=253\to \;w=\frac{l_{x}}{253}\mathrm{and}\;\frac{3920}{16}=245\to \;w=\frac{l_{x}}{245}.$$

## 4. Analysis of the Results and Discussion

**Figure 14:**
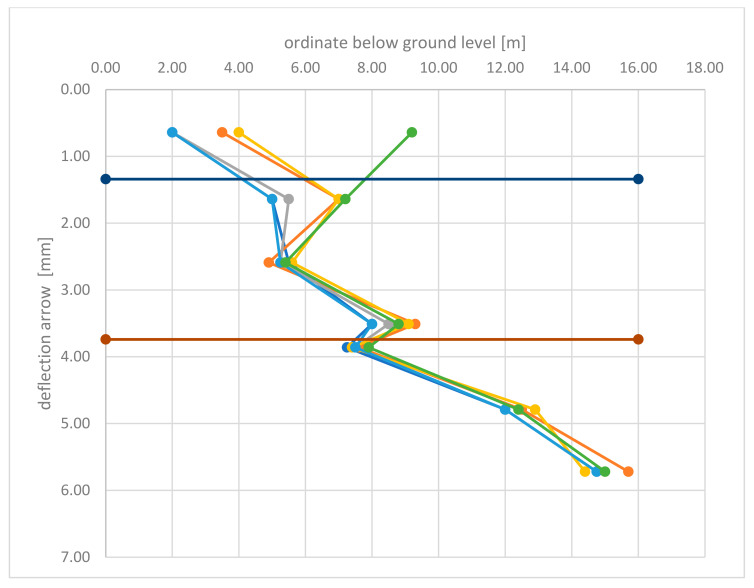
Deflection arrows—Stage-I.

**Figure 15:**
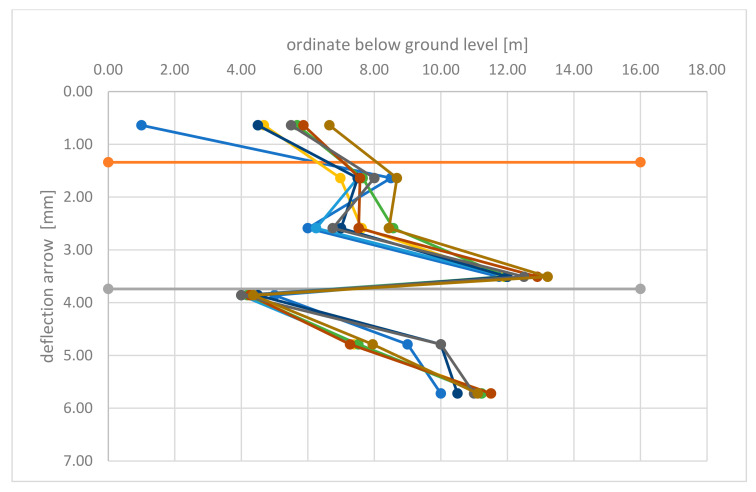
Deflection arrows—Stage-II.

Analysis of the measured deflections, both taken with a patch and using a laser scanner (Table 4, Table 5, Table 6, Table 7, Table 8 and Table 9), showed that the backfill load has no significant effect on the deflections of the lower plate. The maximum values of deflections were recorded in the lower edge of the bottom support plate, 5.72 m below ground level and did not exceed 16 mm for measurement taken with a laser scanner and 15.5 mm for direct measurement taken with a patch. Direct comparison of the deflection arrow values measured with a laser scanner and a patch (Table 8 and Table 9) indicates a high compliance of measurements. The values of measurements of the deflection arrow taken at the same time, with a difference of one to two days between measurements taken with a patch and a laser scanner, for both Stage-I and Stage-II, differed slightly, on average by 0.67 mm, with the value of standard deviation (SD) 1.45 mm (Figure 14 and Figure 15). Greater values of the deflection arrow in individual support plates were recorded in their lower edges, for the middle plate and for the bottom plate. Lower values of the deflection arrow in the upper parts of support plates resulted from the use of strong rib reinforcement in the upper part of the plate (i.e., plate head). Additionally, the middle plate partially overlapped the bottom plate, thus both plates contributed to the load transmission at their contact point. The greater value of deflection 14.4 to 15.7 mm, measured in the lower edge of the bottom plate, occurred probably because the cutting edge of the bottom support plate was less rigid than the rest of the plate surface.

There are few works in the subject literature analysing the results obtained in field experiments as well as analysing measurements taken with one of the most modern technologies, namely laser scanning.

The authors of the paper [76] presented the results of the numerical calculations using the finite element method, which were carried out with the PLAXIS-3D Finite Element computer program. They analysed the value of soil pressure on the steel box cover, acting as an excavation support. These tests included a simulation of the static operation of two steel (or aluminum) excavation covers placed one on the other with a total depth of the excavation equal to 6 m. The finite element method (FEM) was adopted for the analysis and the Mohr-Coulomb constitutive material model (MC) was selected. The results were compared with the empirical diagrams of apparent soil pressure for clay. The comparison showed that the parameters related to the soil and the excavation support structure have a significant impact on the values of received loads and the use of load-bearing capacity of the material from which the support was made. The authors of the work [77] presented the results of experimental field tests, the purpose of which was to examine the value of soil pressure for temporary excavation supports. They provided procedures for instrumentation installation and field testing for two supports of “stacked” excavations, in accordance with the US OSHA regulations, placed in the excavation at a total depth of 6 m. The results of field tests were presented considering the distribution of soil pressure at the excavation depth considering the backfill load and excluding it. The results indicated the possibility of using the tested support system of excavations and ditches in cohesive soils and confirmed the compliance with theoretical results. The authors of the paper [77] found that the results obtained by means of analytical formulas generally underestimated the experimental values for the clay considered in their study.

The effectiveness of applying the finite difference method has been reported in numerous scientific papers. The authors of the article [29] verified the values of deflection experimentally by means of a coordinate measuring arm, comparing them with the values calculated with the finite difference method for hydrostatic thrust acting on the tank. The paper [27] presents calculations for a thermally loaded plate, which were experimentally verified.

Table 10 summarizes the values of deflections in the lower edge of the excavation support (at the bottom of the excavation) obtained from tests carried out with a measurement patch, a laser scanner and calculated using the finite difference method based Tables [73] and a detailed solution with no backfill load.

It can be clearly stated that the compliance of the obtained results was very good. Thus, both the research experiment and the calculations were carried out correctly.

Calculations of the deflection arrow values, which could be used to recommend the formula for the limit deflection of steel plates of temporary excavation supports, were made based on the values of deflection obtained experimentally and numerically. To compare the obtained results, the deflection arrow values were determined based on the material data of the support plate, which should be treated as a limit (permissible).

**Figure 16:**
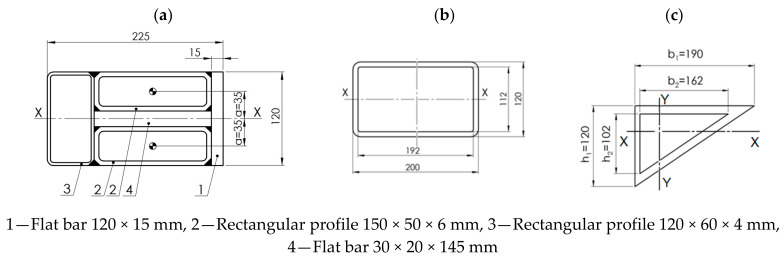
Cross-sections of individual components of the plate, (**a**) head, (**b**) components (rectangular pipe), (**c**) cutting edge.

Calculations were made in two variants for steel S235JR and S355JR, for the plate shown in Figure 3. It was assumed that the fin plate is a freely supported plate loaded with a uniformly distributed load along its entire length (Figure 16).

The moment of inertia for the entire plate (“head” + 10 profiles 200 × 120 × 4 × 4 mm + “cutting edge”) was *I_x_* = 7817.01 cm^4^.

The assumed load capacity of the cross-section taking into account plastic reserve for S235JR steel was $M_{c,\mathrm{Rd}}=M_{\mathrm{pl},\mathrm{Rd}}=\frac{W_{\mathrm{pl},x}}{\gamma_{M0}}\cdot \;f_{y}=33,865.38\;\mathrm{kNcm}$, and for S355JR steel—$M_{c,\mathrm{Rd}}=M_{\mathrm{pl},\mathrm{Rd}}=\frac{W_{\mathrm{pl},x}}{\gamma_{M0}}\cdot \;f_{y}=51,158.34\;\mathrm{kNcm}$. The maximum bending moment for a freely supported beam, uniformly loaded along its entire length, was assumed: $M_{\mathrm{Ed}\;}=0.125\cdot q\cdot l_{x}{}^{2}$.

The maximum plastic load capacity of the cross-section was adopted as the limit value beyond which the steel cross-section plasticization occurs; therefore, the maximum value of the linear load *q* was: for S235JR steel $q={\frac{M_{Ed}}{0.125\cdot l_{x}{}^{2}}}_{}=1.76\;\mathrm{kN}\cdot {\mathrm{cm}}^{-1}$, and for S355JR steel—$q={\frac{M_{Ed}}{0.125\cdot l_{x}{}^{2}}}_{\;}=2.66\;\mathrm{kN}\cdot {\mathrm{cm}}^{-1}$.

By assuming partial load factors, the continuous linear service load was calculated (*q_u_*):

$$q_{u}=\frac{q}{\gamma_{F\;}\cdot \gamma_{M\;}}=\frac{q}{1.5\;\cdot 1.1}:$$

for S235JR steel *q_u_* = $\frac{1.76}{1.5\;\cdot 1.1}$ = 1.067 kN·cm^−1^,
for S355JR steel *q_u_* = $\frac{2.66}{1.5\;\cdot 1.1}$ = 1.61 kN·cm^−1^.

To determine the maximum deflection for the value of plastic resistance of the cross-section, the formula for the deflection arrow was used for a freely supported beam loaded with a load uniformly distributed over its entire length:

$$f=\frac{5\cdot \;q_{u}}{384\cdot \;E\cdot \;I_{x}}\cdot l_{x}^{4\;}$$

where:

*l_x_*—length of the plate (*l_x_* = 392 cm),
*E*—modulus of elasticity for steel (*I* = 210,000 N·mm^−2^) [78],
*I_x_*—moment of inertia (*I_x_* = 7817.01 cm^4^),
*q_u_*—service load.

For S235JR steel:

*f* = $\frac{5\;\cdot 1.067}{384\;\cdot 21000\;\cdot 7817}\cdot 392^{4}=2.00\;\mathrm{cm}$

*f* = 2.00 cm $\to \;\frac{392}{2.00}=196\to f=\frac{1}{196}l_{x}$—the value of deflection was 1/196 of the span.

For S355JR steel:

*f* = $\frac{5\;\cdot \;1.61}{384\;\cdot 21000\;\cdot \;7966}\cdot 392^{4}$ = 3.02 cm

*f* = 3.02 cm $\to \;\frac{392}{3.02}=130\to f=\frac{1}{130}l_{x}$—the value of deflection was 1/130 of the span.

Based on calculations of the deflection arrow, the following results were obtained and are summarised in Table 11.

## 5. Conclusions

The paper presents the results of deflection tests for temporary excavation support plates obtained based on measurements taken on the experimental object in 1:1 scale. This is undoubtedly a great cognitive value of the work. In the subject literature, there are few studies in this field, the results of which are based on real-scale experimental tests and on a real research object. The results obtained during the tests were verified by numerical calculations using the finite difference method.

Based on the experimental tests and numerical analysis, it can be concluded that:

- Deflections measured with a patch and obtained using a laser scanner (Table 8 and Table 9) showed similar values, corresponding to the values resulting from static calculations made with the finite difference method.
- The measured maximum deflections (Table 4, Table 5, Table 6 and Table 7) ranged from 14.40 to 16 mm, and the calculated values were 14.95 and 14.99 mm. The consistency of results is very good.
- Based on the maximum plastic load capacity of the steel fin plate cross-section, the values of permissible deflections were determined. Deflections measured with a patch and a laser scanner were significantly smaller than the values accepted as permissible.
- The analysis of the values of deflections measured both with a patch and a laser scanner (Table 4, Table 5, Table 6 and Table 7) showed that the backfill load does not have a significant effect on the deflection of the lower plate, but it does affect the deflection of the first plate up to a depth of 1.2 m. Deflections of the plate without the backfill load are sometimes greater than deflections with the backfill load recorded for the second and third plate.
- According to PN-EN 13331-1-1:2002 [74], there is no obligation to verify the deflection of support plates of temporary excavations; only the value of the maximum deflection should be provided to users. The authors of the paper believe that this reflects an oversight on the part of the legislator. It would be advantageous if the person conducting construction works (site manager) knew the boundary value of the deflection arrow for excavation support plates, which depends only on the span of the plate, so there is no need to perform any numerical calculations.
- It is recommended, based on Table 11, to assume the limit (maximum) deflection arrow for support plates of temporary excavations at least as $w_{gr}=\frac{L}{130}$, where L is the span of the plate.

The conducted tests, measurements and calculations can be used to verify existing solutions in the subject matter and to improve plate structures in order to ensure greater safety of the manufactured and support plates in use as well as to reduce their production costs.

## Figures and Tables

**Table 1:** Height ordinates of direct measurement points of the deflection arrow taken with a measurement patch.

Measurement No./Support Plate No.	Height Ordinate Below Ground Level
1/plate 1 (upper)	0.64
2/plate 2 (middle)	1.64
3/plate 2 (middle)	2.59
4/plate 2 (middle)	3.51
5/plate 3 (bottom)	3.86
6/plate 3 (bottom)	4.79
7/plate 3 (bottom)	5.72

**Table 2:** Dates of measurements by laser scanning and backfill load.

	Date	Backfill Load [kN·m^−2^]
Stage-I	20 September 2019	0.00
	29 September 2019	15.36
	06 October 2019	26.88
	12 October 2019	38.40
Stage-II	29 October 2019	3.84
	05 November 2019	15.36
	14 November 2019	26.88
	24 November 2019	38.40

**Table 3:** Geotechnical parameters of the soil in the area of the test stand acc. to PN-81/B-03020 [71].

Layer Gap		Soil Type	Compaction Degree	Volumetric Weight	Angle of Internal Friction
from	to		$I_{D}$	*γ*	*φ*_u_
[m]	[m]	[-]	[-]	[kN·m^−3^]	[°]
0.0	0.2	Soil	-	17.00	-
0.2	0.7	MSa	0.74	18.86	34.61
0.7	1.4	MSa	0.45	18.43	32.67
1.4	1.8	MSa	0.57	18.61	33.47
1.8	2.2	Saπ	0.43	17.51	30.15
2.2	2.8	MSa	0.79	18.94	34.94
2.8	3.8	FSa	0.74	18.21	31.70
3.8	5.1	FSa	0.88	18.52	32.40
5.1	5.7	Saπ	0.47	17.60	30.35
5.7	6.0	MSa	0.94	19.16	35.94

**Table 4:** Results of deflection measurements of support plates taken with a patch (Stage-I).

Date		I 24 September 2019	II 25 September 2019	III 26 September 2019	IV 30 September 2019	V 04 October 2019	VI 14 October 2019
Plate	Ordinate of the measurement m below ground level	Backfill load [kN⋅m^−2^]					
		0.00	3.84	15.36	15.36	26.88	38.4
		[mm]	[mm]	[mm]	[mm]	[mm]	[mm]
upper plate	0.64	-	-	-	2.00	2.00	2.00
middle plate	1.64	-	5.50	5.75	5.00	5.50	5.00
	2.59	-	5.00	7.00	5.50	5.25	5.25
	3.51	9.00	9.25	9.75	8.00	8.50	8.00
bottom plate	3.86	7.00	6.75	7.25	7.25	7.50	7.50
	4.79	12.50	12.50	12.00	12.00	12.00	12.00
	5.72	15.50	15.50	15.25	14.75	14.75	14.75

**Table 5:** Results of deflection measurements of plates in mm—manual measurement taken with a patch (Stage-II).

Date		29 October 2019	05 November 2019	12 November 2019	21 November 2019
Plate	m below ground level	Backfill load [kN⋅m^−2^]			
		3.84	15.36	26.88	38.40
upper plate	0.64	1.00	4.50	4.50	5.50
middle plate	1.64	8.50	7.50	7.50	8.00
	2.59	6.00	6.25	7.00	6.75
	3.51	11.75	12.00	12.00	12.50
bottom plate	3.86	5.00	4.00	4.50	4.00
	4.79	9.00	10.00	10.00	10.00
	5.72	10.00	10.50	10.50	11.00

**Table 6:** Results of deflection measurements of excavation support plates for Stage-I (intact soil) obtained with laser scanning.

Date		20 September 2019	29 September 2019	06 October 2019	12 October 2019
Plate	m belowground level	Backfill load [kN⋅m^−2^]			
		0.00	15.36	26.88	38.40
		[mm]	[mm]	[mm]	[mm]
upper plate	0.64	0.30	3.50	4.00	9.20
middle plate	1.64	6.80	7.00	7.00	7.20
	2.59	5.50	4.90	5.60	5.40
	3.51	9.30	9.30	9.10	8.80
bottom plate	3.86	8.10	7.60	7.40	7.90
	4.79	12.60	12.50	12.90	12.40
	5.72	16.00	15.70	14.40	15.00

**Table 7:** Results of deflection measurements of excavation support plates for Stage-II (loosened soil) obtained with laser scanning.

Date		29 October 2019	05 November 2019	12 November 2019	24 November 2019
Plate	m belowground level	Backfill load [kN⋅m^−2^]			
		3.84	15.36	26.88	38.40
upper plate	0.64	4.68	5.67	5.87	6.65
middle plate	1.64	6.98	7.65	7.57	8.68
	2.59	7.62	8.57	7.53	8.44
	3.51	11.90	11.96	12.90	13.21
bottom plate	3.86	4.26	4.18	4.26	4.36
	4.79	7.39	7.52	7.27	7.95
	5.72	11.50	11.23	11.51	11.11

**Table 8:** List of deflection measurements of support plates using a patch and a laser scanner taken for Stage-I. Denominations: P—measurement taken with a patch, S—measurement taken with a laser scanner.

Date		30 September 2019 (Scanner: 29 September 2019)		04 October 2019 (Scanner: 06 October 2019)		14 October 2019 (Scanner: 12 October 2019)	
Backfill load [kN⋅m^−2^]		15.36		26.88		38.4	
		P	S	P	S	P	S
	m belowground level	[mm]					
upper plate	0.64	2.00	3.50	2.00	4.00	2.00	9.20
middle plate	1.64	5.00	7.00	5.50	7.00	5.00	7.20
	2.59	5.50	4.90	5.25	5.60	5.25	5.40
	3.51	8.00	9.30	8.50	9.10	8.00	8.80
bottom plate	3.86	7.25	7.60	7.50	7.40	7.50	7.90
	4.79	12.00	12.50	12.00	12.90	12.00	12.40
	5.72	14.75	15.70	14.75	14.40	14.75	15.00

**Table 9:** List of deflection measurements of support plates using a patch and a laser scanner taken for Stage-II. Denominations: P—measurement taken with a patch, S—measurement taken with a laser scanner.

Date		28 October 2019 (Scanner: 29 October 2019)		05 November 2019		12 November 2019 (Scanner: 14 November 2019)		21 November 2019 (Scanner: 24 November 2019)	
Backfill load [kN⋅m^−2^]		3.84		15.36		26.88		38.40	
		P	S	P	S	P	S	P	S
	m below ground level	[mm]							
upper plate	0.64	1.00	4.68	4.50	5.67	4.50	5.87	5.50	6.65
middle plate	1.64	8.50	6.98	7.50	7.65	7.50	7.57	8.00	8.68
	2.59	6.00	7.62	6.25	8.57	7.00	7.53	6.75	8.44
	3.51	11.75	11.90	12.00	11.96	12.00	12.90	12.50	13.21
bottom plate	3.86	5.00	4.26	4.00	4.18	4.50	4.26	4.00	4.36
	4.79	9.00	7.39	10.00	7.52	10.00	7.27	10.00	7.95
	5.72	10.00	11.50	10.50	11.23	10.50	11.51	11.00	11.11

**Table 10:** Comparison of the values of deflections for support plates using a patch and a laser scanner taken for Stage-I and calculated using Tables and detailed calculations, with the value of backfill load equal to 0.00 kN⋅m^−2^.

The Method of Obtaining the Values of Deflections	The Value of Deflection in the Lower Edge of the Plate, at the Bottom of the Excavation, for Backfill Load 0.00 kN⋅m^−2^
patch (Table 4)	*w* = 15.50 mm
scanner (Table 6)	*w* = 16.00 mm
calculations acc. [73]	*w* = 14.95 mm
detailed FDM calculations	*w* = 14.99 mm

**Table 11:** List of the limit value of the deflection arrow obtained based on experimental tests and calculations.

The Method of Obtaining the Values of Deflection	Calculated Value of the Deflection Arrow (Where *l_x_* Is the Length of the Plate)
Patch (*w*_max_ = 15.50 mm)	$w_{gr}=\frac{l_{x}}{253\;}$
Scanner (*w*_max_ = 16.00 mm)	$w_{gr}=\frac{l_{x}}{245\;}$
Calculations acc. [73] (*w*_max_ = 14.95 mm)	$w_{gr}=\frac{l_{x}}{262\;}$
Detailed FDM calculations (*w*_max_ = 14.99 mm)	$w_{gr}=\frac{l_{x}}{261\;}$
Calculations based on the maximum plastic load capacity for S235JR steel	$w_{gr}=\frac{l_{x}}{196\;}$
Calculations based on the maximum plastic load capacity for S355JR steel	$w_{gr}=\frac{l_{x}}{130\;}$

## Data Availability

Not applicable.
